# Sensor-Based Categorization of Upper Limb Performance in Daily Life of Persons With and Without Neurological Upper Limb Deficits

**DOI:** 10.3389/fresc.2021.741393

**Published:** 2021-10-20

**Authors:** Jessica Barth, Keith R. Lohse, Jeffrey D. Konrad, Marghuertta D. Bland, Catherine E. Lang

**Affiliations:** ^1^Program in Physical Therapy, Washington University in St. Louis, St. Louis, MO, United States; ^2^Program in Occupational Therapy, Washington University in St. Louis, St. Louis, MO, United States; ^3^Department of Neurology, Washington University in St. Louis, St. Louis, MO, United States

**Keywords:** upper extremity, accelerometry, cluster analysis, rehabilitation, outcomes assessments, stroke

## Abstract

**Background:** The use of wearable sensor technology (e. g., accelerometers) for tracking human physical activity have allowed for measurement of actual activity performance of the upper limb (UL) in daily life. Data extracted from accelerometers can be used to quantify multiple variables measuring different aspects of UL performance in one or both limbs. A limitation is that several variables are needed to understand the complexity of UL performance in daily life.

**Purpose:** To identify categories of UL performance in daily life in adults with and without neurological UL deficits.

**Methods:** This study analyzed data extracted from bimanual, wrist-worn triaxial accelerometers from adults from three previous cohorts (*N* = 211), two samples of persons with stroke and one sample from neurologically intact adult controls. Data used in these analyses were UL performance variables calculated from accelerometer data, associated clinical measures, and participant characteristics. A total of twelve cluster solutions (3-, 4-, or 5-clusters based with 12, 9, 7, or 5 input variables) were calculated to systematically evaluate the most parsimonious solution. Quality metrics and principal component analysis of each solution were calculated to arrive at a locally-optimal solution with respect to number of input variables and number of clusters.

**Results:** Across different numbers of input variables, two principal components consistently explained the most variance. Across the models with differing numbers of UL input performance variables, a 5-cluster solution explained the most overall total variance (79%) and had the best model-fit.

**Conclusion:** The present study identified 5 categories of UL performance formed from 5 UL performance variables in cohorts with and without neurological UL deficits. Further validation of both the number of UL performance variables and categories will be required on a larger, more heterogeneous sample. Following validation, these categories may be used as outcomes in UL stroke research and implemented into rehabilitation clinical practice.

## Introduction

The use of wearable sensor technology (e.g., accelerometers) for tracking human movement has allowed for efficient measurement of activity of the upper limb (UL) in daily life (1–6). Accelerometry has become an established, valid and reliable methodology to directly measure performance of UL activity in daily life in neurologically intact adults (7, 8) and adults with stroke (9–13). Per the World Health Organization International Classification of Functioning, Disability and Health (ICF) model (14), activity *performance*, defined as what a person does in the unstructured, free-living environment, is a different but related construct to the *capacity* for activity (i.e., functional capacity), which is measured by standardized assessments in the structured clinical or laboratory setting. Clinicians and researchers typically assess a person's functional capacity for activity in the structured clinic or laboratory environments with standardized assessments. However, people seek out rehabilitation services because they want to be able to perform better in their daily lives (15), and improvements in UL capacity seen in the clinic do not necessarily translate to improvements in UL performance in daily life (13, 16–19). Therefore, assessment of UL activity performance in an individual's unstructured, free-living environment is critical to evaluating effectiveness of rehabilitation services and determining if the services provided have achieved the goal of improving performance in daily life.

Data extracted from bilateral, wrist-worn accelerometers can be used to quantify variables measuring different aspects of UL performance in one or both limbs. These variables collectively inform clinician scientists about the real-world activity performance. The numerous variables calculated from accelerometers measure different aspects of UL performance, such as: (1) duration (7, 20); (2) magnitude (12, 21, 22); (3) variability (12, 23); (4) symmetry or laterality (3, 7, 9); and (5) quality of movement (6, 24–26). Each UL performance variable conveys slightly different information about the collective nature of UL use, with a single variable providing only part of the picture (6). Furthermore, some variables are narrowly distributed in neurologically-intact (adult controls) individuals (e.g., use ratio, an index of duration of activity of one limb vs. the other), while other variables are widely distributed (e.g., bilateral magnitude, a measure of magnitude of bilateral UL activity) (3). Thus, multiple variables quantifying different aspects of movement along with heterogeneous distributions of those variables can make it difficult to interpret UL performance data for clinical decision-making.

One reason wearable sensor technology (e.g., accelerometry) for measurement of UL performance has remained largely confined to rehabilitation research with limited ventures into clinical practice is because the current output from accelerometers is not easily accessible for rehabilitation professionals (4). A potential solution to the multi-variable problem would be the formation of categories (or groups) of UL performance in daily life. If there were natural groupings that occur among multiple UL performance variables calculated from accelerometry data (27), then these groupings could help to facilitate clinical decision making and implementation of UL performance data into routine rehabilitation care. In other biomedical science fields, formation of categories which encompass multi-dimensional measures have facilitated clinical decision making for persons with health conditions such as, spinal cord injury (28), heart failure (29, 30), and chronic obstructive pulmonary disease (31).

The purpose of this study, therefore, was to identify categories of UL performance in daily life in adults with and without stroke using data from previously collected cohorts. Cluster analyses were performed with variables of UL performance calculated from 24 h accelerometer recordings from three cohorts, two samples of persons with stroke and one from neurologically-intact adult controls. We hypothesized that at least three categories (low, medium, and high) of UL performance would be identified across the UL performance variables quantified by accelerometer data, spanning the possible ranges of UL performance in daily life. We also anticipated that the emerging categories would group individuals with similar ranges of the performance variables and provide a simpler method to interpret UL performance in daily life for clinicians and persons with health conditions whom they treat.

## Methods

This study analyzed accelerometer data from adults from three previous cohorts, using the same accelerometry methodology (32). Data used in these analyses were UL performance variables calculated from accelerometer data over 1 day, associated clinical capacity measures, and participant characteristics.

### Participants

The three cohorts in this analysis include; (1) people with stroke (*stroke cohort 1, n* = *57*) from a prospective, observational, longitudinal cohort tracking UL change over time (19); (2) people with chronic stroke (*stroke cohort 2, n* = *78*) who participated in a clinical trial (33); and (3) a sample of neurologically-intact adults (*adult controls, n* = *76*) of similar age, race, ethnicity, and socioeconomic status of persons in the clinical trial (*stroke cohort 2*) (7). All participants provided signed informed consent to participate in the individual studies. Inclusion and exclusion criteria for each sample are described elsewhere [*stroke cohort 1* (19), *stroke cohort 2* (33), and *adult controls*(7)]. In general, persons in the stroke cohorts had documented UL motor impairments and diminished functional capacity as measured by the Action Research Arm Test (ARAT) (34, 35) at the time of the study enrollment. UL motor severity ranged from mild to severe, as indicated by the National Institute of Health Stroke Scale (NIHSS) (36) arm item scores of 1–4. Persons with stroke had to be able to follow two-step commands to enroll, and were enrolled even if they had other, mild, stroke-induced, non-motor deficits such as hemispatial neglect, aphasia, or mild cognitive impairment. Neurologically intact community-dwelling older adults had to be willing to participate and be able to follow two-step commands. Combining the three cohorts provided a broad sampling of UL performance variables. With respect to power analyses, there is no agreed upon sample required for a cluster analysis (37, 38), however the combined cohorts yield a sample size of over 200 individuals, which was deemed sufficient to proceed with a cluster analysis (39).

### Data Collection

UL performance was captured using data from bilateral, wrist-worn accelerometers (7, 8, 40–42). A single time point was chosen for participants in each of the three cohorts. In *stroke cohort 1* (assessments from 2 to 24 weeks post-stroke), data from the latest assessment time point available between weeks 6 and 24 were used in the analysis, since UL performance appears to stabilize between 3 and 6 weeks post-stroke (19, 43). In *stroke cohort 2* (assessments at baseline and weekly for 8 or more weeks), data from the earliest available assessment time point was used in the analysis. Data points later than the baseline (when baseline was unavailable) were included because UL performance did not change as a result of this treatment (18, 33). The *adult control* cohort completed a single assessment in the cross-sectional study and this time point was used (8).

### Upper Limb Performance Variables

Participants wore the Actigraph GT3X-BT or GT9X-Link accelerometers on both wrists for the three cohorts, with methods described previously (32). Briefly, tri-axial acceleration data are sampled at 30 Hz for 24 or more hours continuously. Once the accelerometers were returned to the lab, data were uploaded, visually inspected, and processed using Actilife 6 (Actigraph Corp., Pensacola, FL) proprietary software. For most variables, data were band-pass filtered (0.25 and 2.5 Hz) and down sampled into 1-s epochs with ActiLife proprietary software, where each second is the sum of the 30 Hz values in that second and converted to activity counts (1 count = 0.001664 g). For a few variables (see Table 1), calculations were done directly on the 30 Hz data (6, 24–26). Similar to previous work (7, 12, 19, 21, 43), accelerometry data was processed using custom written software in MATLAB (Mathworks, Inc., Natick, MA) to calculate UL performance variables which qualify various aspects of UL activity in everyday life. Table 1 presents the 12 UL performance variables included in the analysis along with their description and the source of accelerometer data for calculation (1 vs. 30 Hz). The variables independently measure duration, magnitude, variability, symmetry and quality of movement of one or both ULs.

**Table 1:** Upper limb performance variables.

**Upper limb performance variable name**	**Description**	**Data source**	**Included in final solution**
**Duration**			
Hours of paretic/non-dominant limb activity (7, 8)	Time, in hours, that the paretic/non-dominant limb is moving.	1 Hz	**✓**
Hours of non-paretic/dominant limb activity (7, 8)	Time, in hours, that the non-paretic/dominant limb is moving.	1 Hz	**✓**
Isolated paretic/non-dominant limb activity (8)	Time, in hours, that the paretic/non-dominant limb is moving, while the non-paretic/dominant limb is still.	1 Hz	
Isolated non-paretic/dominant limb activity (8)	Time in hours that the non-paretic/dominant limb is moving, while the paretic/non-dominant limb is still.	1 Hz	
**Magnitude**			
Median acceleration of paretic/non-dominant limb^*^(3, 8, 21)	Magnitude of accelerations of the paretic/non-dominant limb, in activity counts or gravitational units.	1 Hz	**✓**
Bilateral magnitude^*^(3, 8, 21)	Intensity, or magnitude of accelerations of movement across both arms, in activity counts.	1 Hz	
**Variability**			
Acceleration variability of paretic/non-dominant limb activity^*^(12, 23)	Standard deviation of the magnitude of accelerations across the paretic/non-dominant limb, reflecting the variability of paretic/non-dominant limb movement, in activity counts.	1 Hz	**✓**
**Symmetry**			
Use ratio^†^ (7, 9, 40)	Ratio of hours of paretic/non-dominant limb movement, relative to hours of non-paretic/dominant limb movement.	1 Hz	**✓**
Magnitude ratio^†^ (8, 12, 23)	Ratio of the magnitude of paretic/non-dominant UL accelerations relative to the magnitude of the non-paretic/dominant UL accelerations. This ratio reflects the contribution of each limb to activity, expressed as a natural log.	1 Hz	
**Quality of movement**			
Jerk asymmetry index (26)	Ratio of the average jerk magnitude between the paretic/non-dominant limb and the non-paretic/dominant limb. Higher jerk represents less smooth movement, and an index of 0 represents similar smoothness of movement in the paretic/non-dominant and non-paretic/dominant limbs. Values are bounded between −1 and +1.	30 Hz	
Spectral arc length of paretic/non-dominant and non-paretic/dominant limb (6, 24, 25)	A measure of movement smoothness that quantifies movement intermittencies independent of the movement's amplitude and duration.	30 Hz	
	Longer spectral arc lengths are reflective of less smooth or less coordinated movement in either the paretic/non-dominant or non-paretic/dominant limb respectively	30 Hz	

* *Variables that are quantified in activity counts, computed by the Actilife proprietary software such that 1 activity count = 0.001664 g*.

† *For persons with stroke, ratios are paretic to non-paretic, while for neurologically-intact adults, ratios are non-dominant to dominant*.

### Analysis

All data were analyzed in R (version 4.0.1), an open source statistical computing program. A *k-*means hypothesis-free cluster analysis was used to determine categorizations of UL performance indexed by accelerometer variables in samples of persons with stroke and neurologically intact adults (adult controls). A cluster analysis is a robust statistical algorithm that groups similar objects into sub-groups called clusters (27, 44, 45), with identified clusters becoming the categories of UL performance. The end point is a set of clusters where individuals within each cluster are more similar to each other, on average, than they are to other members of the other clusters formed (44). A *k-*means method was chosen over other methods (e.g., hierarchical clustering or partial around the medeoid) to use an iterative approach to qualitatively explore the effect of adding more input variables and increasing the number of clusters on the dataset used in the analysis (45, 46).

First, several steps were completed prior to the cluster analysis. The dataset of UL performance variables were standardized (using z-scores) as each variable is on a different measurement scale (e.g., hours, counts, and ratios). Then, a Hopkins statistic was calculated to determine if pursing a cluster analysis on these data was appropriate. The Hopkins statistic ranges from 0 to 1, and values >0.5 indicate clusters exist in the dataset (47). The distributions of all 12 UL performance variables and pairwise spearman scatterplots of variables with both strong and weak relationships were examined using the GGally package (48). Distributions and scatterplots were used to understand the relationships between UL performance variables in preparation for additional analyses and for later simplification of the cluster solutions that emerged.

Second, a principal component analysis (PCA) was conducted using the factoextra package on datasets that included 12, 9, 7, or 5 of the UL performance variables (49). Principal components can be thought of as the underlying dimensions of the individual UL performance variables (45). PCAs were calculated including all 12 performance variables, then variables were systematically eliminated to exclude the variables that are complex to calculate (e.g., used 30 vs. 1 Hz data) and the variables with less straight forward clinical interpretation. Scree plots were examined for each of the models (5, 7, 9, and 12 UL performance variables) to determine how many principal components explained variance in the UL performance variables. Further, we examined the loadings of the input variables on each of the resulting PCs.

Third, different numbers of clusters were evaluated and the solutions were calculated using the NbClust and clusertend packages (50, 51). A *k-*means cluster analysis expects the number of clusters to be specified prior to the analysis. Thus, we started with 3-clusters as a reasonable solution to produce clusters of low, medium and high UL performance. There are multiple statistical methods for determinizing the optimal number of clusters. We evaluated potential solutions using: (1) the elbow method (52), (2) the silhouette method (53), and (3) the gap statistic (27). Although there was no clear single “elbow” where adding clusters led to diminishing returns in variance explained, these methods indicated that 3-, 4-, and 5-cluster solutions were progressively better explanations of the data (see section Results). Thus in the interests of parsimony, we focused on these three different cluster sizes in subsequent analyses.

A total of 12 cluster solutions (3-, 4-, or 5-clusters with 12, 9, 7, and 5 input variables) were calculated to systematically eliminate UL performance variables to create the most parsimonious solution (50, 51). The most complex model was calculated first (including all 12 performance variables) for a 3-, 4-, and 5-clusters. The second most complex model included 9 UL performance variables, excluding the three variables calculated from the 30Hz data that are proposed to measure quality of UL activity (6, 24–26) (see Table 1). These variables were removed because they are more complex to calculate, have not been validated in clinical populations (22), and did not add relevant information to the analysis. For the 7 and 5 input variable models, the decision was made to maintain at least one performance variable from each of the other four aspects of UL performance (duration, magnitude, variability and symmetry) to capture the dimensionality of UL performance in daily life. Variables that were simpler to calculate (1 vs. 30 Hz) and interpret were retained over those that required more complex calculations and/or are more difficult to interpret for ease of eventual integration into rehabilitation clinics (4). For example, both the bilateral magnitude and the median acceleration of the paretic/non-dominant limb activity quantify the magnitude or intensity of UL activity. These two variables are highly correlated to each other and the loadings from the PCA indicate that these two variables had moderate, positive loadings on PC1, primarily. For the 5 variable solution, the median acceleration of the paretic/non-dominant limb was selected to remain because it had a higher contribution to PC1 than the bilateral magnitude and it is a simpler variable to calculate and interpret.

Fourth, we examined model fit metrics for each of the 12 solutions calculated to avoid overfitting as additional variables and clusters were added. The total variance explained by the models were extracted for each of the cluster-variable solutions (3-, 4-, or 5-clusters with 12, 9, 7, and 5 input variables). Models that had a higher % of total explained variance were deemed to have a better model-fit (45). Additionally, a multivariate analysis of variance (MANOVA) was calculated to re-fit the cluster classifications (3-, 4-, and 5-clusters) to the multi-dimensional space of all the UL performance variables (5, 7, 9, and 12 variables). This allowed for the Akaiki information criterion (AIC) to be extracted to compare the model-fit for each of the cluster solutions with respect to the variables included (45). As the AIC imposes a penalty for additional model parameters, selecting the model with the lowest AIC value helps avoid overfitting and improve generalizability.

Fifth, the means and ranges of the UL performance variables, concordance, and UL capacity (e.g., ARAT score) were computed for each cluster in the final solution. Given statistically significant omnibus effects from the multivariate analyses described above, univariate ANOVAs were computed to determine how the means of the UL performance variables differed from each other across the clusters (alpha = 0.05) (54, 55). *Post-hoc* comparisons (using a Tukey HSD correction) of each cluster to other clusters for five different performance variables were calculated (alpha = 0.05). Additionally, we looked at how the input cohorts (*stroke cohort 1, stroke cohort 2, adult controls*) were distributed across the cluster solutions.

Finally, coxcomb charts were created. Coxcomb charts are a two-dimensional chart type designed to plot one or more series of values over multiple quantitative variables. The 5 UL performance variables are divided into equally segmented wedges on the radial chart. The area of each individual wedge is proportional to the magnitude of the score on that dimension. Coxcomb charts were created from the standardized performance variables to provide a visual representation of the UL performance variable scores in each cluster both at the group and individual level.

## Results

A sample of 211 participants were included in the analyses. Demographic and participant characteristics for the three cohorts are provided in Table 2. UL capacity was measured by the ARAT and indicated that both stroke cohorts were and had moderate deficits in UL functional capacity.

**Table 2:** Demographics and participant characteristics of the three cohorts.

**Variable**	**Stroke cohort 1** **(***n*** = 57)**	**Stroke cohort 2** **(***n*** = 78)**	**Adult controls** **(***n*** = 76)**
**Age**, years	66.5 ± 8.8	59.7± 10.9	54.3 ± 11.3
**Sex**, female	42% (24)	35% (27)	51% (39)
**Race**			
African American	40% (23)	47% (36)	59% (44)
Caucasian	58% (33)	51% (40)	41% (30)
Asian	2% (1)	1% (1)	NA
Other		1% (1)	
**Time post-stroke**, weeks	24 (6–24)	52 (21–960)	NA
**Hand dominance**, right	82% (47)	88% (68)	82% (62)
Concordance^*^	42% (24)	51% (40)	NA
**Action research arm test** ^†^	22.46 ± 20.76	31.3 ± 11.9	NA

*Values are Mean ± SD or Percentage (n) except for Time-post-stroke which are median (range)*.

* *Concordance is where dominant limb = paretic limb*.

† *Action Research Arm Test is a measure of UL functional capacity. Higher scores are better, with a maximum total score of 57 indicating normal performance*.

The Hopkins statistic was *H* = 0.78, indicating that clusters exist in the sample. Table 3 summarizes the range of solutions evaluated including 12, 9, 7, and 5 UL performance variables in either a 3-, 4-, or 5-cluster solutions. Across the different numbers of input variables, two principal components explained the majority of the variance, PC1 and PC2. There were similar loadings of the input variables onto these principal components, regardless of the number of variables entered. Interestingly, adding more performance variables (e.g., 12 vs. 5) was associated with both PC1 and PC2 explaining less of the total variance (see the first column of Table 3). Thus, across different numbers of input dimensions, the number of principal components was relatively stable. PC1 and PC2 appeared to be explaining similar variance in all models. We therefore proceeded with including only 5 input variables. When including 5 UL performance variables, the first principal component (PC1) explained the most variance (76.4%) and was comprised of variables that all had moderate to strong, positive loadings, including; paretic/non-dominant hours, median acceleration of paretic/non-dominant limb activity, acceleration variability of paretic/non-dominant limb activity and the use ratio. The second principal component (PC2) explained less variance (17.6%) and was comprised of primarily the non-paretic/dominant hours, a single variable that had a strong, negative loading. See Supplementary Table 1 for the loadings of all factors of PC1 and PC2 for the final chosen solution.

**Table 3:** Selection of clusters based on variance explained and model-fit.

**Number of variables**	**Variance explained by each PC**		**Total variance by # of clusters**			**AIC value by # of clusters**		
	**PC1**	**PC2**	**3**	**4**	**5**	**3**	**4**	**5**
12	57.4%	13.1%	53%	58%	62%	2, 683.7	2, 442.2	2, 149.8
9	68.5%	16.5%	64%	69%	73%	1, 426.4	1, 114.7	879.2
7	75.6%	14.1%	70%	76%	79%	734.3	452.9	228.0
5	76.4%	17.6%	68%	75%	79%	475.6	229.1	44.7

*Explained variance is presented in %. Values closer to 100% indicate greater variation explained*.

*AIC, Akaike's Information Criterion. A lower AIC value indicates a better model when the clusters were used as predictor variables in multivariate ANOVAs based on the different outcome variables (of 12, 9, 7, and 5 dimensions)*.

**Figure 1:**
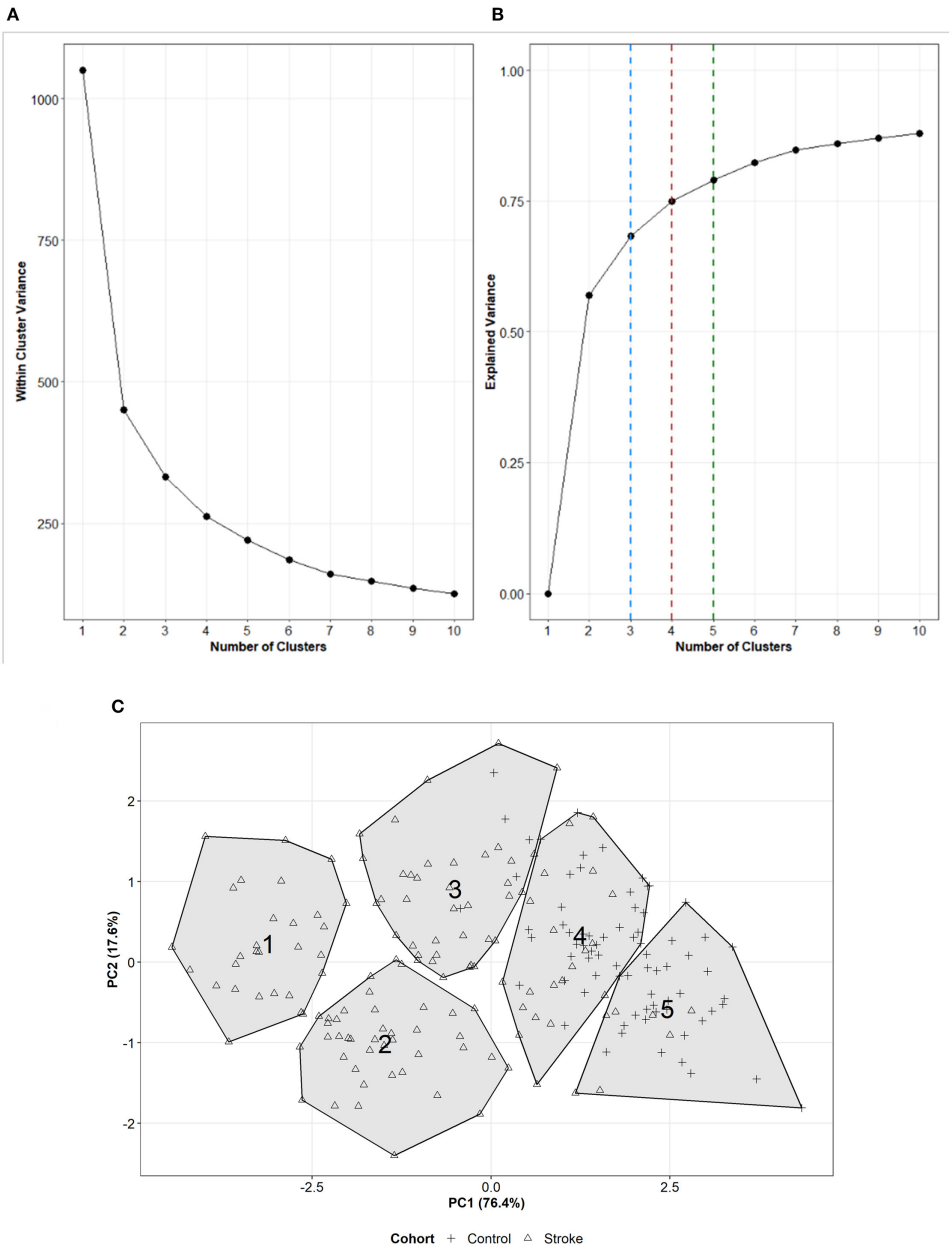
**(A)** Scree plot representing how the within-cluster variance changes as increasing numbers of clusters are formed with 5 UL performance variables. **(B)** Line plot representing how the total explained variance changes with increasing numbers of clusters on dataset including 5 UL performance variables. The dashed lines represent the total variance explained for a 3- (blue), 4- (red), or 5- (green) cluster solution. **(C)** Visual representation of the 5-clusters with 5 UL performance variables across dimension 1 (x-axis) and dimension 2 (y-axis). The cluster number is presented in the location of the centroid of each cluster. The shape of the point within the cluster represents the if a participant was from a stroke (triangle) or control (+ sign) cohort.

**Figure 2:**
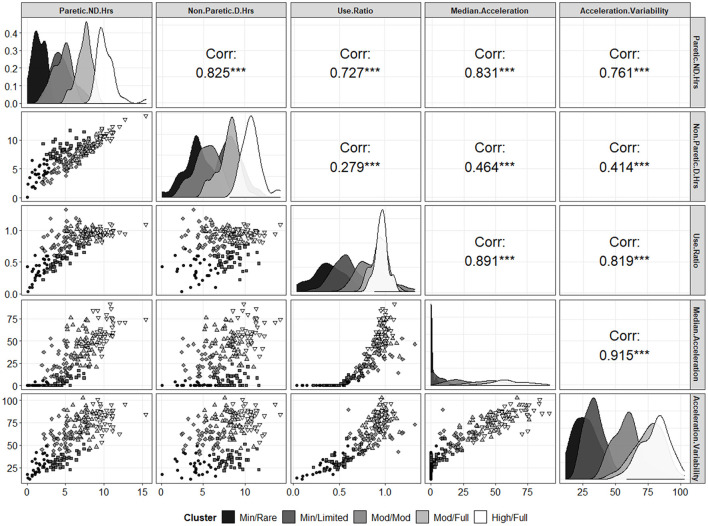
Scatterplot matrix of the 5 input variables as a function of the 5 different clusters. The diagonal shows density plots (i.e., the univariate distribution) of each input variable as a function of the different clusters. The lower left panels show the bivariate distributions for each pair of variables with the point shapes and gray scales corresponding to the different clusters (see legend). The upper right panels show the Spearman rank order correlations for each pair of variables (on the whole, ignoring clusters). ^***^p < 0.001.

Across the models with differing numbers of UL performance variables, a 5-cluster solution explained the most overall total variance when compared to a 3- or 4-cluster solution as seen in the middle portion of Table 3 and visually in Figures 1A,B (including 5 performance variables). We then examined several metrics to determine how many clusters were appropriate for the 5-variable solution. Figure 1A supports that there are ≥ at least two clusters in this dataset and the flattened slope on Figure 1A indicates that the reduction of within-cluster variance is minimal and there are no further improvements after 5-clusters for this dataset. We therefore explored a 3-, 4-, and 5- cluster solutions. Figure 1B displays the effect of increasing numbers of clusters on the total explained variance when including 5 UL performance variables and confirms that a 5-cluster solution explains more total variance than the 3- or 4-cluster solutions. Examining the AIC values seen in the last three columns of Table 3, also confirmed that a 5-cluster solution produced the best model fit compared to the 3- and 4-cluster solutions across the different number of input variables (5, 7, 9, and 12 UL performance variables). Although each solution was statistically feasible, the chosen final solution was 5-clusters, from 5 UL performance variables including: (1) hours of use of paretic/non-dominant limb; (2) hours of use of non-paretic/dominant limb; (3) median acceleration of paretic/non-dominant limb; (4) acceleration variability of paretic/non-dominant limb activity; and (5) use ratio. Figure 1C presents the location of the 5-clusters across the two dimensional space. Dimension 1 (x-axis) is the first principal component and dimension 2 (y-axis) is the second principal component. The two clusters with the lowest overall UL performance are represented by clusters numbered 1 and 2 with the highest in number 5. Figure 2 shows a scatterplot matrix of how the 5 input variables relate to each other and to the 5-clusters.

**Figure 3:**
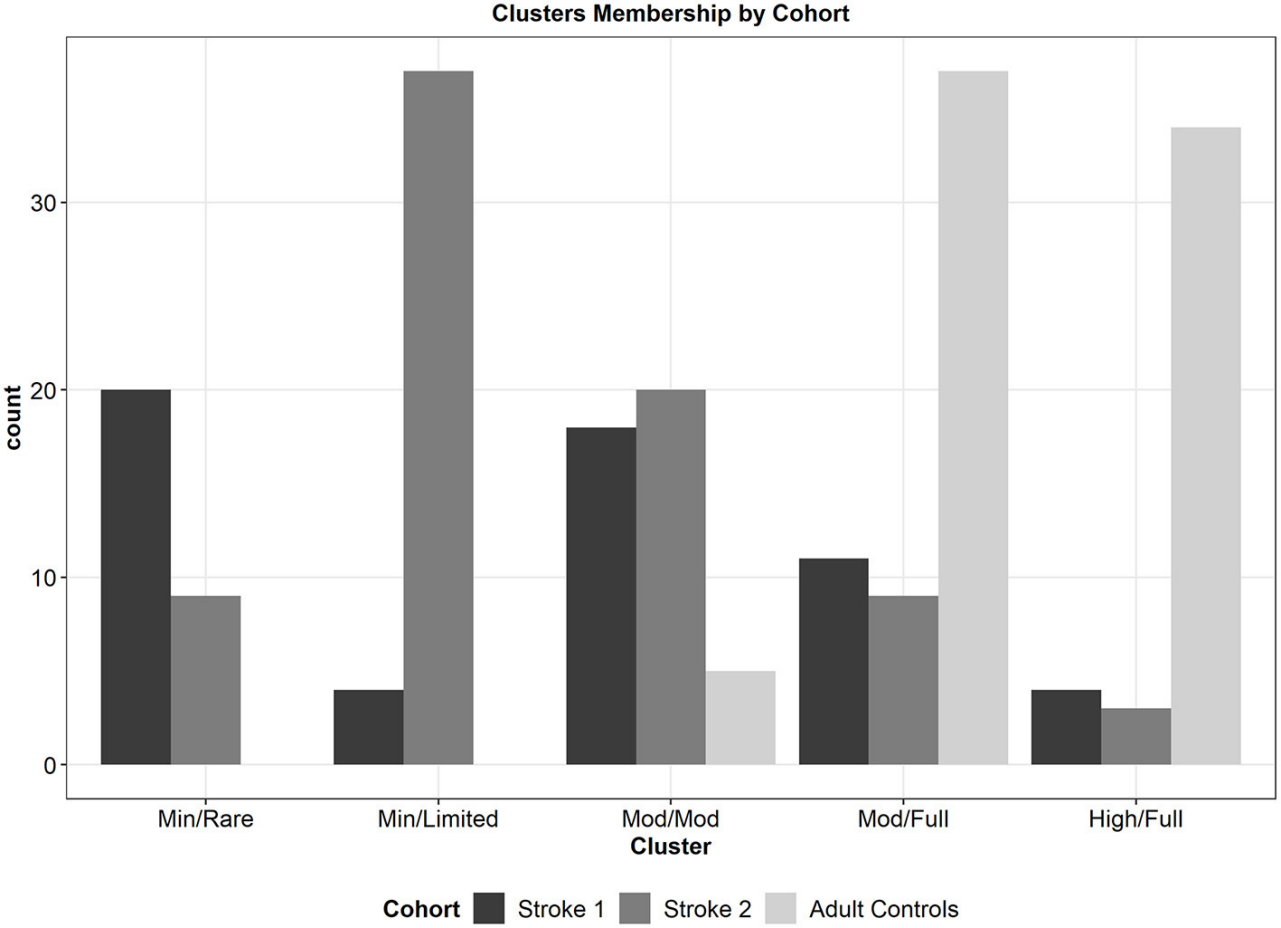
Bar plot of the counts of participants from each of the 3 cohorts that separated into the 5-clusters. The two clusters with the lowerst overall UL performance are comprised of persons from the stroke cohorts only. The cluster with moderate UL performance contains primarily persons with stroke and a few neurologically intact adult controls. The two clusters with the highest overall UL performance include primarily neurologically intact adult controls, as well as persons with stroke.

The means and ranges of each UL performance variable, percentage concordant, and UL capacity for each of the 5-clusters in the final solution are presented in Table 4. The clusters are presented with the “lowest” overall UL performance within the first column and the “highest” overall UL performance in the last column. The 5-clusters become categories of UL performance and are named based on a synthesis of information from other publications that have described UL performance in daily life (45, 56, 57) not on the underlying PCA dimensions. The cluster names were chosen as intuitively as possible and represent the overall amount of UL activity and integration of the ULs into daily life activities (see Discussion for further interpretation). We refer to these clusters/categories as: *(1) Minimal Activity/Rare Integration; (2) Minimal Activity/Limited Integration; (3) Moderate Activity/Moderate Integration; (4) Moderate Activity/Full Integration; and (5) High Activity/Full Integration*. The cluster with the lowest UL performance is the *Minimal Activity/Rare Integration*, this cluster has the lowest mean values on variables that quantify duration, magnitude and variability of UL activity. People in this cluster use their non-paretic UL ~2.5 times more than their paretic UL and have little to no magnitude or variability of their paretic UL activity in daily life. People in the *Minimal Activity/Limited Integration* cluster use both the paretic and non-paretic limb for more overall hours than the *Minimal Activity/Rare Integration* cluster, but the non-paretic limb is still active twice as much as the paretic UL. Additionally, people in this cluster have slightly higher mean values on performance variables that quantify both the magnitude and variability of the paretic limb when compared to the *Minimal Activity/Rare Integration* cluster. Both of these clusters have little integration of the ULs into activity, as suggested by a mean use ratio below 0.50 the *Minimal Activity/Rare Integration* cluster and a mean use ratio just above 0.50 in the *Minimal Activity/Limited Integration* cluster. The cluster with overall, moderate UL performance is the *Moderate Activity/Moderate Integration* cluster. In this cluster, people have more symmetrical UL use compared to the two lower clusters, which is reflected in the in the use ratio (0.85) and the mean values of both duration variables (4.5 vs. 5.3 h). People in this cluster have moderate values on variables that quantify both the magnitude and variability of paretic/non-dominant limb activity. The two clusters with the highest overall UL performance are the *Moderate Activity/Full Integration and the High Activity/Full Integration* clusters. These clusters have progressively higher mean values of variables quantifying duration, magnitude and variability of UL activity with those in the *High Activity/Full Integration* cluster having the highest mean values compared to the other clusters. Both of these clusters however, have similar mean values of the use ratio, which is approaching 1.0 indicating that people in these two clusters have relatively equal contributions of both ULs. Interestingly, if only the use ratio was used to examine these two clusters it could be assumed that they are relatively equal, but the other variables show they are not. The two clusters with the highest overall UL performance also had the highest % of people with concordant stroke. It is also noteworthy that participants within each of the 5 clusters have wide, overlapping ranges of UL capacity, as indicated by the mean and ranges of ARAT scores in the bottom row of Table 4, consistent with the premise that UL capacity and UL performance are different, but related constructs. Figure 3 presents how the three included cohorts separated into the 5-clusters. The two clusters with the lowest overall UL performance (*Minimal Activity/Rare Integration* and *Minimal Activity/Limited Integration)* are comprised of only persons from the stroke cohorts. The cluster with moderate UL performance (*Moderate Activity/Moderate Integration)* contains mostly people with stroke but there are also a few neurologically intact adult controls in this cluster too. The two clusters with the highest overall UL performance (*Moderate Activity/Full Integration* and *High Activity/Full Integration)* contains the neurologically intact adult controls and some persons with stroke. Finally, there was a statistically significant omnibus effect of cluster in each of the univariate ANOVAs for the 5 UL performance variables (*p*-values for each variable < 0.001). Note that not all clusters were statistically different from all other clusters in each variable, based on *post-hoc t*-tests. However, this speaks to the multivariate nature of the cluster analyses; across all dimensions, these clusters group similar to observations together, but along any single dimension there will likely be overlap in the neghiboring clusters.

**Table 4:** Means (ranges) of UL performance and capacity variables by cluster.

**Variable name**	**Minimal activity/** **rare integration** **(***N*** = 29)**	**Minimal activity/** **limited integration** **(***N*** = 41)**	**Moderate activity/** **moderate integration** **(***N*** = 43)**	**Moderate activity/** **full integration** **(***N*** = 57)**	**High activity/** **full integration** **(***N*** = 41)**
**Duration**					
Paretic/ND Hrs	1.5 (0.0–2.8)	4.6 (2.1–8.0)	4.5 (1.9–6.5)	7.4 (5.2–9.1)	10.2 (8.6–15.5)
Non-paretic/D Hrs	4.1 (0.1–6.7)	8.4 (6.2–11.6)	5.3 (2.4–8.0)	8.0 (5.1–11.0)	10.7 (8.5–14.2)
**Magnitude**					
Median acceleration paretic/ND (counts)^*^	0 (0–6)	5 (5–24)	25 (7–53)	47 (21–76)	61 (33–92)
**Variability**					
Acceleration variability of paretic/ND (counts)^*^	27.3 (11.9–49.4)	34.8 (21.6–57.3)	58.9 (40.0–89.3)	75.9 (46.5–102.6)	80.3 (53.0–100.8)
**Symmetry**					
Use ratio	0.38 (0.04–0.70)	0.55 (0.22–0.78)	0.85 (0.60–1.32)	0.94 (0.75–1.15)	0.96 (0.81–1.10)
**Additional data about the clusters**					
Concordance^†^	38% (11)	39% (16)	50% (19/38)	70% (14/20)	57% (4/7)
Action research arm test^‡^	18.5 (0–43)	27.8 (6–57)	45.3 (22–57)	48.4 (33–57)	44.1 (24–55)

* *Data are reported in activity counts computed by the Actilife proprietary software, such that 1 activity count = 0.001664 gravitational units (g)*.

† *Dominant limb = paretic limb, computed for persons in stroke. Percentage is expressed relative to only persons with stroke, not controls, in the upper three categories*.

‡ *Action Research Arm Test is a measure of UL functional capacity. Higher scores are better, with a maximum total score of 57 indicating normal capacity*.

**Figure 4:**
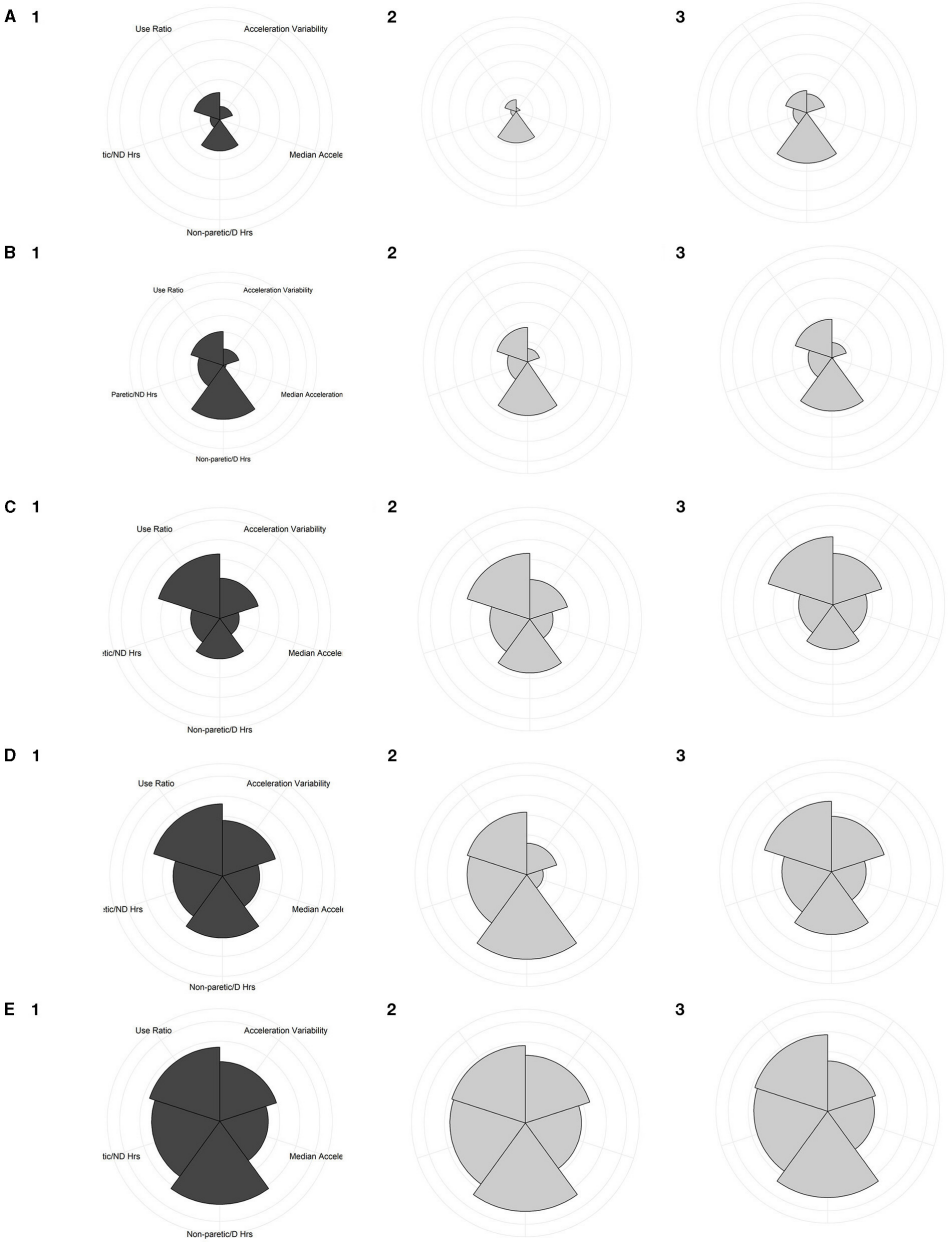
Coxcomb charts of the five clusters, illustrating the contribution of the UL performance variables on a standardized scale. The first column plots group data, while the 2nd and 3rd columns plot individual participant examples. **(A)** *Minimal Activity/Rare Integration* cluster; (A1) group chart of people within this cluster; (A2) is a person from stroke cohort 1, ARAT = 4; and (A3) is a person from stroke cohort 2, ARAT = 10. **(B)** *Minimal Activity/Limited Integration* cluster; (B1) group chart of people within this cluster; (B2) a person from stroke cohort 2, ARAT = 10; and (B3) a person from stroke cohort 1, ARAT = 6. **(C)** *Moderate Activity/Moderate Integration* cluster; (C1) group chart of people within this cluster; (C2) a person from stroke cohort 1, ARAT = 36; and (C3) a person from the adult controls. **(D)** *Moderate Activity/Full Integration* cluster; (D1) group plot for this cluster; (D2) a person from stroke cohort 2, ARAT = 42; and (D3) a person from the adult controls. **(E)** *High Activity/Full Integration cluster;* (E1) group chart of people within this cluster; (E2) a person from stroke cohort 1, ARAT = 55; and (E3) a person from the adult controls.

Figure 4 presents the group and individual coxcomb charts for each of the 5-clusters. The rows (A, B, C, D, E) are presented in order of increasing overall UL performance, with group data in the first column in dark gray and then individual examples of people in that cluster in columns two and three (A-E, numbered 2 and 3 respectively) in light gray. Each of the 5 standardized UL performance variables are represented by wedges within the plot, and the area of the wedge reflects the standardized value on that single variable. Figures 4A,B present the two clusters with the lowest overall UL performance (*Minimal Activity/Rare Integration and Minimal Activity/Limited Integration)*, the wedges in these two clusters are small with the exception of the non-paretic/D hours of use, indicating that people in these two clusters use their non-paretic UL out of proportion to their paretic UL. As you move down each row from *Minimal Activity/Rare Integration* (Figure 4A) to *High Activity/Full Integration* (Figure 4E) one can see that the wedges get larger and begin to fill more area of the radial plot, however some variables are still out of proportion to the others as seen in Figures 4C,D. By the final group plot in Figure 4E1, the wedges for each variable span the largest area and almost form a perfect circle, compared to the clusters with lower UL performance (Figures 4A,B), indicating people in this cluster have the highest values across all 5 performance variables.

## Discussion

In a large sample of persons with and without neurological UL deficits, we used a *k-*means cluster analysis with multiple UL performance variables, captured *via* accelerometry, to derive a 5-cluster categorization that included 5 UL performance variables. Two principal components explain most of the variance in the input variables and 5-clusters explained the most total variance and had the best model fit. In this 5-cluster solution, two groups with what might be considered “normal” UL performance (*Moderate Activity/Full Integration and High Activity/Full Integration*) emerged, as indicated by the presence of many neurologically intact adult controls in those categories. One category in the middle had moderate UL performance (*Moderate Activity/Moderate Integration*), while two categories had low, overall UL performance (*Minimal Activity/Rare Integration* and *Minimal Activity/Limited Integration)*. The names of each of the 5 categories were chosen for their overall UL activity and integration, with the future goal that these categories could be evaluated for their application to other clinical populations, not just persons with stroke.

The 5-category solution from 5 UL performance variables, derived from this statistical analysis, leads to a clinically-logical interpretation of UL performance in daily life. In this analysis we purposefully included three cohorts of persons with and without stroke in order to capture a wide range of the variables, extracted from accelerometer data, that quantify different aspects of UL performance in daily life. In Figure 3, the two categories with the highest overall UL performance (*Moderate Activity/Full Integration* and *High Activity/Full Integration*) contain most of the neurologically intact adult controls indicating that people without neurological impairments display a wide range of UL activity that can be considered unimpaired or normal. This is important because these people have integrated their ULs, as indicated by the use ratio variable, but people in these categories have different levels of overall UL activity, ranging from moderate to high UL activity. This is not unusual when we consider the wide range of activities and behaviors of people (58–60). For example, when walking performance is quantified by pedometers, neurologically-intact adults walk symmetrically but present with a wide range of variability in the total number of steps-per-day that can all be considered “normal” walking performance (58, 61–65). Based on the current results, it appears that people without neurological UL impairments similarly display a wide range of UL activity that can also be considered unimpaired or normal. For example, two neurologically intact older adults may have very different activities of daily living and leisure activities (e.g., swimming vs. knitting) but would both be considered to have “normal” UL performance. In other efforts to categorize UL activity, some groups have found four categories (54, 55, 57), and others have found six (56). These analyses however tended to examine only the separation of UL activity of persons with stroke. In this analysis, the goal was not to form categories to differentiate between those who had a stroke and those who did not. Instead, the goal was to categorize people based on their overall UL use in daily life. In the 5-category solution here, we see that the two categories with the lowest UL activity and integration are comprised of only persons with stroke, but there are also people with stroke in the three categories with the highest overall UL performance too. This is a positive finding, showing that some people with stroke use their ULs similarly to neurologically intact adults. Persons with stroke who ended up in the two categories with the highest overall UL performance have likely experienced either full recovery of their ULs following their stroke, or have figured out how to use the wide range of capacity that they have to integrate their paretic limb and be active in daily life (19). An example of this is shown in Figure 4E2 which is an individual from *stroke cohort 1* who ended up in the *High Activity/Full Integration* category.

Categories of UL performance have tremendous research and clinical potential. Within other biomedical science fields, formation of categories which encompass multi-dimensional measures have facilitated clinical decision making for persons with health conditions (see section Introduction). Specific to rehabilitation, categories of ambulation (based on the capacity measure of walking speed) have been validated, shown to be sensitive to change (66–68), used to set goals in clinical practice, and have been used as a primary outcome in a Phase III clinical trial (69). In that trial, the primary outcome was the percentage of people who changed (leaped) to a higher ambulation category after the intervention. The identified categories of UL performance that emerged in this analysis could be useful for future trials of persons with UL impairments following subsequent, future validation studies. Categories that emerged in this analysis have stratified participants into groupings with similar overall UL performance, representing a profile of arm activity in daily life (38, 55, 70). Individuals within each category have similar ranges of each performance variable included (e.g., duration, magnitude, variability and symmetry) that formed the 5-clusters. Interestingly, in this analysis people with stroke within each of the five clusters display a wide range of UL capacity across the clusters. Additionally, more people in the two clusters with highest overall UL performance have concordant stroke compared to the three clusters with lower UL performance. These findings are consistent with prior work indicating that people with concordant stroke (dominant limb = paretic limb) tend to have differences in the patterns of UL use (56, 71) and experience better recovery (19). One can envision that these categories could be used in future trials to analyze smaller subsets of individuals based on their UL category and to better understand how UL performance variables quantify change during rehabilitation therapy.

From a clinical perspective, the categories that emerged offer the future opportunity to transition measurement of UL performance in daily life for persons receiving UL rehabilitation away from the current confines of rehabilitation research labs, and into standard of care (4, 72). The results of this analysis are a first step in simplifying measurement of UL performance in daily life by exploring the underlying structure in the set of observed variables (73). A future option could be to offer a user-friendly, software package to rehabilitation clinicians that would calculate the 5 UL performance variables included in this analysis from data extracted from bilateral wrist-worn accelerometers. Based on a person's values across the variables, a category of UL performance could be determined and used to communicate current UL performance and used to set goals for future UL performance. Based on the aspects of movement (duration, magnitude, variability, symmetry) selected to form the categories, it is possible that these categories could be highly relevant for many clinical conditions affecting UL performance in daily life, not just those with stroke. Just as with mobility, there are plenty of biological and psychological reasons why people could have limited UL performance in daily life (58, 74, 75). Thus, the names selected for each category might be applicable to other clinical populations that have similar or different UL impairments and capacity limitations, beyond the typical asymmetrical deficit which is a major aspect of stroke UL movement (3).

### Limitations

There are a few limitations to consider when interpreting the results of this study. First, the three cohorts used in this analysis generated a sample of over 200 people with stroke and neurologically intact adult controls. While our sample size was large and had wide distributions of each UL performance variable, validation on another large, independent sample is needed for generalization and implementation into clinical practice. Future studies, including people with other clinical diagnoses beyond stroke are needed in order to understand how the number of UL performance variables and subsequently the number of clusters generalize to other populations. Second, the *Moderate Activity/Moderate Integration* category is less straightforward to understand than the other four categories that emerged in this analysis. This category is comprised primarily of persons from both stroke cohorts, however there are a few neurologically intact adult controls who ended up in this category as well. Unfortunately, we do not have enough information about other cognitive, socioeconomic, physical, emotional or behavioral reasons why these few people without neurological UL impairments ended up in this category with reduced overall UL activity and integration. This category specifically will need to be externally validated in a larger sample.

### Conclusions

The present study identified 5 categories of UL performance in a combined cohort of neurologically impaired and unimpaired adults. These categories can be formed with a minimum of 5 UL performance variables, extracted from bilateral wrist-worn accelerometers that span the possible ranges of UL activity and integration. Further validation of both the number of UL performance variables and categories will be required on a larger, more heterogenous sample. Following validation, these categories may be used as outcomes in UL stroke research and implemented into rehabilitation therapies.

## Data Availability Statement

Publicly available datasets were analyzed in this study. This data can be found here: https://simtk.org/projects/referentaccdata.

## Ethics Statement

The studies involving human participants were reviewed and approved by Human Research Protection Office, Washington University. The patients/participants provided their written informed consent to participate in this study.

## Author Contributions

CL: financial. JB, JK, KL, MB, and CL: manuscript preparation. JB, JK, KL, and CL: data analysis. JB and MB: data collection. JB and CL: study design. All authors contributed to the article and approved the submitted version.

## Funding

This work was funded by the US National Institutes of Health R01HD068290, T32HD007434, and TL1TR002344.

## Conflict of Interest

The authors declare that the research was conducted in the absence of any commercial or financial relationships that could be construed as a potential conflict of interest.

## Publisher's Note

All claims expressed in this article are solely those of the authors and do not necessarily represent those of their affiliated organizations, or those of the publisher, the editors and the reviewers. Any product that may be evaluated in this article, or claim that may be made by its manufacturer, is not guaranteed or endorsed by the publisher.
