# Supplementary material for: Sensor-Based Categorization of Upper Limb Performance in Daily Life of Persons With and Without Neurological Upper Limb Deficits
Source: Front Rehabil Sci. 2021 Oct 20;2:741393. doi: 10.3389/fresc.2021.741393 (PMC8979497; doi:10.3389/fresc.2021.741393)
Supplement: Supplementary file 1 [file Table_1.DOCX]

Supplementary Table 1

| **Variable Name** | **PC1 Loadings** | **PC2 Loadings** |
| --- | --- | --- |
| Paretic/ND Hrs | 0.48 | -0.31 |
| Non-paretic/D Hrs | 0.34 | -0.78 |
| Median acceleration paretic/ND (counts)* | 0.45 | 0.39 |
| Acceleration variability of paretic/ND (counts)* | 0.48 | 0.22 |
| Use Ratio | 0.47 | 0.30 |
| **Total Variance Explained** | 76.4% | 17.6% |
